# Purine Nucleoside Phosphorylase Targeted by Annexin V to Breast Cancer Vasculature for Enzyme Prodrug Therapy

**DOI:** 10.1371/journal.pone.0076403

**Published:** 2013-10-03

**Authors:** John J. Krais, Olivier De Crescenzo, Roger G. Harrison

**Affiliations:** 1 Bioengineering Center and the School of Chemical, Biological and Materials Engineering, University of Oklahoma, Norman, Oklahoma, United States of America; 2 Stephenson Cancer Center, Health Sciences Center, University of Oklahoma, Oklahoma City, Oklahoma, United States of America; Stanford University, United States of America

## Abstract

**Background and Purpose:**

The targeting of therapeutics is a promising approach for the development of new cancer treatments that seek to reduce the devastating side effects caused by the systemic administration of current drugs. This study evaluates a fusion protein developed as an enzyme prodrug therapy targeted to the tumor vasculature. Cytotoxicity would be localized to the site of the tumor using a protein fusion of purine nucleoside phosphorylase (PNP) and annexin V. Annexin V acts as the tumor-targeting component of the fusion protein as it has been shown to bind to phosphatidylserine expressed externally on cancer cells and the endothelial cells of the tumor vasculature, but not normal vascular endothelial cells. The enzymatic component of the fusion, PNP, converts the FDA-approved cancer therapeutic, fludarabine, into a more cytotoxic form. The purpose of this study is to determine if this system has a good potential as a targeted therapy for breast cancer.

**Methods:**

A fusion of *E. coli* purine nucleoside phosphorylase and human annexin V was produced in *E. coli* and purified. Using human breast cancer cell lines MCF-7 and MDA-MB-231 and non-confluent human endothelial cells grown *in vitro*, the binding strength of the fusion protein and the cytotoxicity of the enzyme prodrug system were determined. Endothelial cells that are not confluent expose phosphatidylserine and therefore mimic the tumor vasculature.

**Results:**

The purified recombinant fusion protein had good enzymatic activity and strong binding to the three cell lines. There was significant cell killing (*p*<0.001) by the enzyme prodrug treatment for all three cell lines, with greater than 80% cytotoxicity obtained after 6 days of treatment.

**Conclusion:**

These results suggest that this treatment could be useful as a targeted therapy for breast cancer.

## Introduction

Fludarabine (9-β-D-arabinofuranosyl-2-fluoroadenine 5′-monophosphate, 2-Fluoro-ara-AMP, F-ara-AMP), a cytotoxic purine nucleoside analogue, is an approved treatment for chronic lymphocytic leukemia [1], [2], [3], [4]. Fludarabine has undergone numerous clinical trials for treatment of both solid tumors and leukemia; however success was hindered by dose limiting factors including myelosuppression and neurotoxicity [4], [5].

More recently, fludarabine has become the subject of study as the prodrug for enzyme prodrug systems as therapies for cancers such as glioma [6], prostate [7], [8], bladder [9], and liver [10]. The *Escherichia coli* enzyme purine nucleoside phosphorylase (PNP) cleaves the ribose-1-phosphate group from fludarabine, resulting in 2-fluoroadenine [11], [12] which inhibits protein, RNA, and DNA synthesis [13]. Fludarabine is not a substrate for the human PNP. 2-fluoroadenine toxicity to cancer cells occurs at a concentration several orders of magnitude below that required for the same effect from fludarabine [14], which allows for the systemic administration of fludarabine significantly below problematic levels while improving the cytotoxic effect at the site of the tumor. *In vivo* PNP gene delivery studies used intraperitoneal fludarabine doses ranging from 37.5 mg/kg/day [7], [10] to 450 mg/kg/day [15] in mouse models. Minimal effect was attained by fludarabine alone, but results were positive with the conversion to 2-fluoroadenine by the enzyme prodrug therapies. No signs of systemic toxicity or negative side effects were reported at these doses in either the groups with fludarabine or fludarabine with PNP. One advantage of this enzyme prodrug therapy is that there is a significant bystander effect of the 2-fluoroadenine generated against both proliferating and non-proliferating cells [13]. The bystander effect results from the ability of 2-fluoroadenine to freely diffuse across cell membranes, eliminating the need for PNP to be present in each individual cell. This property helps to alleviate transfection efficiency problems of studies using suicide gene-directed enzyme prodrug therapies (GDEPT) [8] and viral-directed enzyme prodrug therapies (VDEPT) [6], [7], [10].

**Figure 1:**
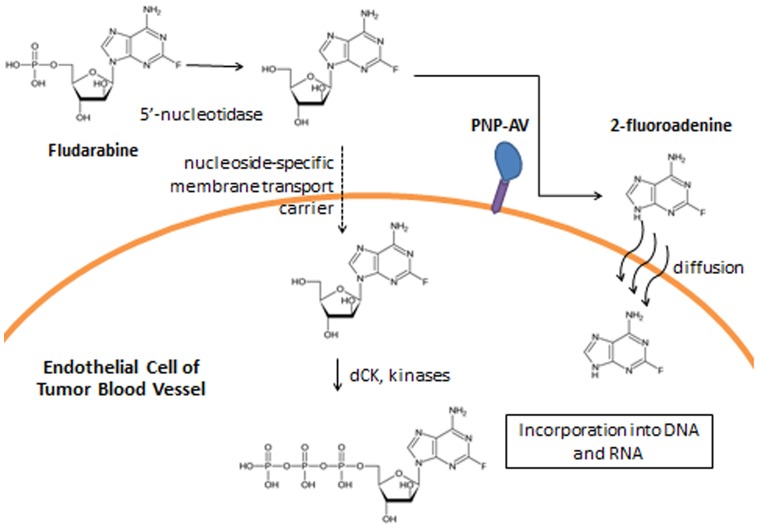
PNP-AV mechanism of action. Fludarabine is converted in serum to a dephosphorylated form by a 5′nucleotidase [37]. PNP attached to the cell surface via annexin V and phosphatidylserine binding then cleaves the ribose-1-phosphate group, resulting in 2-fluoroadenine [11], [12]. The freely diffusible molecule enters the cell and inhibits protein, RNA, and DNA synthesis [13]. Nucelotide-specific membrane transport carriers transport the dephosphorylated form across the cell membrane, where it is then phosphorylated into a cytotoxic triphosphate [3], [37].

An alternative enzyme prodrug approach is to target the PNP enzyme to the tumor with a targeting moiety. We have created a fusion protein of *E. coli* PNP and human annexin V (AV), referred to as PNP-AV. Annexin V binds to phosphatidylserine (PS) expressed externally on tumor cells [16], [17], [18] and endothelial cells of tumor vasculature, but not normal vascular endothelial cells [19], [20]. We have used other enzymes fused to annexin V, and the resulting fusion proteins have been shown to maintain binding properties and successfully target breast cancer cells [21], [22]. This approach to an enzyme prodrug system allows for systemic administration of the fusion protein, with accumulation of PNP in the tumor as a result of AV targeting of the tumor vasculature and cancer cells. After clearance of PNP-AV from the bloodstream, fludarabine would be administered systemically so that 2-fluoroadenine is generated at the surface of the tumor vasculature endothelial cells. The 2-fluoroadenine generated would be expected to have a cytotoxic effect on the endothelial cells and tumor cells, as it would be carried by permeation and diffusion across the vascular wall. Fludarabine is not a substrate of human nucleoside phosphorylases [11], so the conversion to 2-fluoroadenine will not occur in normal tissue. The mechanism of treatment is summarized in Fig. 1.

In the present study, the objective was to produce and evaluate the efficacy *in vitro* of a PNP-AV fusion protein in the context of an enzyme prodrug therapy for the treatment of breast cancer. Protein production was successful, and the binding stability and cytotoxicity of the PNP-AV enzyme prodrug therapy were characterized for estrogen receptor-positive breast cancer MCF-7 cells, estrogen receptor-negative breast cancer MDA-MB-231 cells (a triple-negative breast cancer), and endothelial HAAE-1 cells grown under conditions to mimic the tumor vasculature.

## Materials and Methods

### Construction of Recombinant Expression Plasmid

The PNP gene was provided by Dr. Joanne Turnbull of the Department of Chemistry and Biochemistry of Concordia University (Montreal, Canada). The AV gene was obtained from Dr. Stuart Lind of the University of Colorado (Denver, CO). Both genes were amplified separately with the Expand High Fidelity PCR system from Roche Applied Sciences (Madison, WI). The oligonucleotide PCR primers were synthetically produced by Integrated DNA Technologies (Coralville, IA) and are displayed below. The bold regions on the primers indicate the sequences complementary to the genes. BamHI restriction enzyme sites are shown as the boxed regions with the cut sites indicated. The underlined segment in the PNP antisense primer is a linker region.

PNP gene sense primer:

5′-GAC GAC GAC AAG ATG CCC **GCT ACC CCA CAC ATT AAT GCA G**- 3′

PNP gene antisense primer:

5′-CGC G|GA TCC AGA ACC GGA GCC **CTC TTT ATC GCC CAG CAG AAC**-3′

Annexin V sense primer:

5′-CGC G|GA TCC **GCA CAG GTT CTC AGA GGC**-3′

Annexin V antisense primer:

5′-GA GGA GAA GCC CGG **TTA GTC ATC TTC TCC ACA GAG C-3**′

The PCR products were purified using a QIAquick PCR purification kit (Qiagen, Valencia, CA) and separately digested with BamHI restriction enzyme (New England Biolabs, Ipswich, MA). The digested genes were purified and ligated with T4 DNA ligase (New England Biolabs). The fusion gene was amplified via PCR with the following sense and antisense primers:

Fusion gene sense primer:

5′-CGC T|CT AGA ATG **GCT ACC CCA CAC ATT AAT GCA G-3**′

Fusion gene antisense primer:

5′-CGC C|TCGAG *CGG ACC CTG GAA CAG AAC TTC CAG* **GTC ATC TTC TCC ACA GAG CAG C**-3′

The bold regions indicate the complementary sequences for the start of the PNP gene for the sense primer and the end of the AV gene on the antisense primer. Restriction enzyme sites were incorporated, shown as the boxed regions with the cut site indicated. The sense strand site is for Xba1 and the antisense strand site is for Xho1. The antisense strand also includes a cleavage site for the protease HRV-3C, shown in italics, to allow for separation of the protein from the C-terminal His-tag using immobilized metal affinity column chromatography (IMAC).

The PCR products were again purified using a QIAquick PCR purification kit, run on an agarose gel, and extracted with a QIAquick gel extraction kit from Qiagen. A restriction enzyme digest was performed on the purified PNP-AV gene and pET 303/CT-His vector (Invitrogen, Grand Island, NY) with Xho1 and Xba1 (New England Biolabs). Ligation was performed with T4 DNA ligase. Plasmids were sent to Oklahoma Medical Research Foundation (Oklahoma City, OK) for sequence verification following transformation and culture of NovaBlue Gigasingles competent cells (EMD Chemicals, Gibbstown, NJ). *E. coli* BL21(DE3) cells (EMD Chemicals) were then transformed and cultured for the protein expression.

### Recombinant PNP-AV Expression and Purification

Recombinant PNP-AV was produced from a 1 liter culture of BL21(DE3) cells and purified using IMAC with immobilized Ni^2+^, according to the procedure of Zang et al. [23]. The fusion protein, collected in the IMAC elution, was incubated with HRV-3C protease to cleave the His-tag from the protein. A second IMAC was performed to remove the protease, which also has a His-tag for separation purposes, and uncleaved PNP-AV. Samples were analyzed using SDS-PAGE with Coomassie blue staining [24], and purity was determined with a densitometric analysis performed with ImageJ software.

### Determination of Protein Content and Enzyme Activity Assay

The Bradford assay (Bio-Rad, Hercules, CA) with bovine serum albumin standards was used for determination of protein content. PNP activity was determined according to the quality control procedure from Sigma [25]. The assay was performed with a 300 µL reaction volume of 90 mM potassium phosphate monobasic at pH 7.4, 0.25 mM inosine, 0.1 U xanthine oxidase, and PNP enzyme with tests performed at 1.7 µg/ml and 0.17 µg/ml. All chemicals were obtained for the PNP activity assay from Sigma-Aldrich (St. Louis, MO). Absorbance at 293 nm was measured every 7 seconds for 5 minutes with a Bio-Tek Synergy HT microtiter plate reader (Winooski, VT), and the slope was determined. The change in absorbance per min for a blank sample was subtracted from the change in absorbance per min of the PNP sample and divided by 12 (the millimolar extinction coefficient of uric acid, a product of the xanthine oxidase catalyzed reaction) and by the concentration of PNP to obtain a result in U/mg. One unit (U) is defined as the quantity of enzyme required for the phosphorolysis of 1.0 µmol of inosine to hypoxanthine and ribose 1-phosphate per minute at pH 7.4 and 25°C.

### Cell Culture

Human breast cancer cell lines MDA-MB-231 and MCF-7 were obtained from American Type Culture Collection (Manassas, VA), and human HAAE-1 aortic endothelial cells were from Coriell Cell Repositories (Camden, NJ). Cell lines were acquired within the past year, expanded, and multiple aliquots were frozen in liquid nitrogen. No cells were used beyond the sixth passage. All cells were maintained in medium containing 10% FBS, penicillin (100 U/ml), and streptomycin (100 µg/ml). MDA-MB-231 cells were grown in Leibovitz’s L-15 medium supplemented with 2 mM L-glutamine at 37°C with no additional CO_2,_ as recommended by the ATCC. MCF-7 cells were cultured in Eagle’s minimum essential medium containing Earle’s balanced salt solution, non-essential amino acids, 2 mM L-glutamine, 1 mM sodium pyruvate, 1.5 g/L sodium bicarbonate and supplemented with 0.01 mg/ml human insulin. HAAE-1 cells were grown in flasks and plates coated with 0.1% gelatin in F-12 K medium with 2 mM L-glutamine and 1.5 g/L sodium bicarbonate and supplemented with 0.03 mg/ml endothelial cell growth supplement and 0.1 mg/ml heparin. MCF-7 and HAAE-1 cells were grown at 37°C with 5% CO_2_.

### Protein Binding Strength and Stability Assays

The dissociation constant to characterize binding of PNP-AV to cells was determined as previously described [21]. This procedure involves fixing the cells with 0.25% glutaraldehyde when they are 80–85% confluent, followed by incubating with biotinylated PNP-AV and then washing. Bound PNP-AV is quantitated by incubation with streptavidin-horseradish peroxidase, washing, incubation with the chromogenic substrate *O*-phenylenediamine and hydrogen peroxide, and measurement of absorbance at 450 nm. At each concentration of PNP-AV, the specific binding was obtained by subtracting the non-specific binding (no CaCl_2_ present, 5 mM EDTA added) from the total binding (2 mM CaCl_2_ added).

The stability of binding of PNP-AV to cells was determined as previously described [21]. In this procedure, the Alamar Blue assay [26] was performed as described in the cytotoxicity methods section on separate sets of cells at 0, 1, 2, and 3 days to determine cell viability, followed by fixing the cells and a determination of the binding.

### Binding Visualization

MCF-7 cells were grown on a coverslip and fixed at 80–85% confluence according to the procedure for determining binding strength. Fixation of cells with glutaraldehyde preserves cell morphology while allowing the exposure of membrane binding sites [27]. Afterwards, the cells were incubated at 37°C for 1 h with biotinylated PNP-AV in growth medium with 2 mM Ca^2+^. After washing, streptavidin-conjugated green Alexa Fluor 488 (Invitrogen) was added to the cells at 5 µg/ml for 30 min in order to visualize biotinylated PNP-AV on the membrane surface (streptavidin does not internalize into formerly viable cells after fixation [28]). Cells were counterstained with 5 µg/ml Hoechst 33258 blue dye (Sigma-Aldrich) for nucleic acids for 30 min and with 1 µg/ml CellMask Deep Red plasma membrane stain (Invitrogen) using three 5 min incubations followed by washes. The coverslip was attached to the slide with fluoro-gel with TES buffer (Fisher Scientific, Hampton, NH). Slides were viewed immediately following preparation with a Leica Microsystems SP8 confocal laser scanning microscope (Buffalo Grove, IL) in both the x-y and x-z planes.

### In vitro Cytotoxicity

The cytotoxicity assays were performed over 6 days as two 3-day cycles in 24-well plates under standard culture conditions with calcium supplemented medium (2 mM Ca^2+^). On first day of each cycle, day 0 and day 3, cells were incubated with 100 nM PNP-AV for 2 h at 37°C. The plates were washed, and medium containing varying concentrations of fludarabine (VWR, Radnor, PA) was added. A control of fludarabine with no fusion protein and a control of 2-fluoroadenine (Thermo Fisher Scientific, Waltham, MA) were also included in the study. The medium containing fludarabine or 2-fluoroadenine was replaced daily for the two subsequent days of each cycle. An Alamar Blue assay was performed to determine viability on days 0, 2, 4, and 6 with a 4-hour incubation of cells at 37°C with 10% Alamar Blue reagent (Invitrogen). Fluorescence was measured using a mictotiter plate reader with an excitation wavelength of 530 nm and emission wavelength of 590 nm after the solutions had been transferred to an opaque 96-well plate.

### Data Analysis

Assays were performed in triplicate and a one-way ANOVA with a Tukey-Kramer multiple comparisons test was used with the cytotoxicity analysis. Prism 5 software (GraphPad, La Jolla, CA) was used to perform the calculations.

## Results

### Protein Expression and Purification

**Figure 2:**
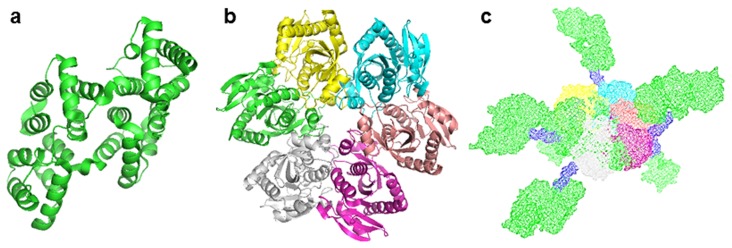
PNP-AV, PNP, AV protein models. Images generated with The PyMOL Molecular Graphics System, Version 1.2r3pre, Schrödinger, LLC with data files obtained through the Protein Data Bank Europe. (a) Image of human annexin V generated from the crystal structure [38]. (b) Image of *Escherichia coli* PNP [39]. **c** Mesh image of a model of PNP-AV hexamer.

Cultures have yielded an average of 60 mg fusion protein per liter of culture. The size of the PNP-AV monomers was confirmed with SDS-PAGE to be 65 kDa as expected based on the known monomeric PNP size of 26 kDa [29], [30], AV size of 36 kDa, and amino acid linker size of 3 kDa. Densitometric analysis determined a purity of >93%. The activity of PNP was determined to be 35 U/mg PNP-AV. Models of the fusion protein and both components are shown in Fig. 2.

### Binding Strength and Stability

**Figure 3:**
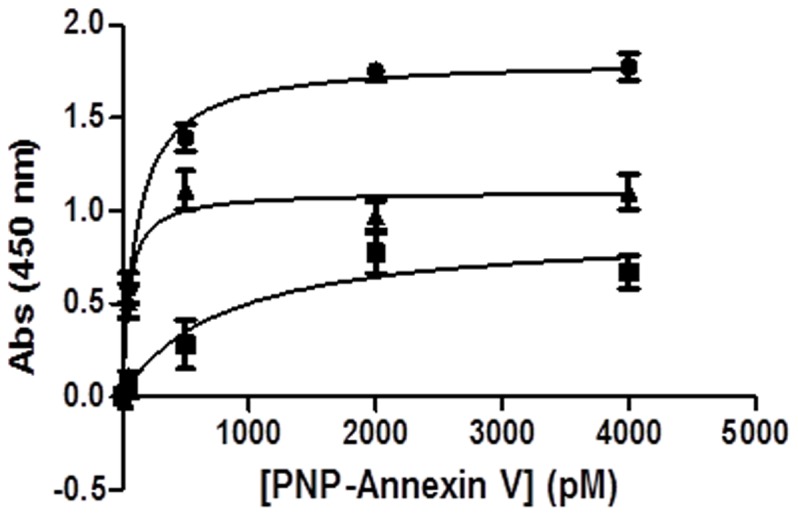
MCF-7 dissociation constant binding data. Specific binding (▴) was determined by subtracting total binding (•) in calcium supplemented medium from nonspecific binding (▪) in calcium deficient medium. Binding was quantified with biotinylated protein and HRP-conjugated streptavidin, developed with OPD. Data are presented as mean ± SE (*n = *3).

A typical equilibrium binding result is shown in Fig. 3 for the binding of PNP-AV to MCF-7 breast cancer cells. The non-specific binding, obtained in the absence of Ca^+2^, is subtracted from the total binding to obtain the specific binding. We previously determined that endothelial cells that are not confluent when cultured *in vitro* expose PS [22]; therefore hydrogen peroxide was not used to induce exposure of PS. It has been reported that cancer cells express PS *in vitro* [16], [17], [18]. The dissociation constant (*K_d_*) for each cell line was obtained from the specific binding data using Prism 5 software to give the results shown in Table 1. The *K_d_* values, which are all below 100 pM, indicate that the binding of PNP-AV to these cells is relatively strong. The binding strength assay was also performed with endothelial cells that were confluent, and no *K_d_* value could be obtained since the specific binding was indistinguishable from the non-specific binding.

**Table 1:** Dissociation constant (*K_d_*) of PNP-AV binding to cells.

Cell line	Dissociation constant (*K_d_*) ± SE (n = 3)
HAAE-1	18.3 pM ±16.4 pM
MCF-7	51.6 pM ±18.0 pM
MDA-MB-231	75.3 pM ±52.3 pM

**Figure 4:**
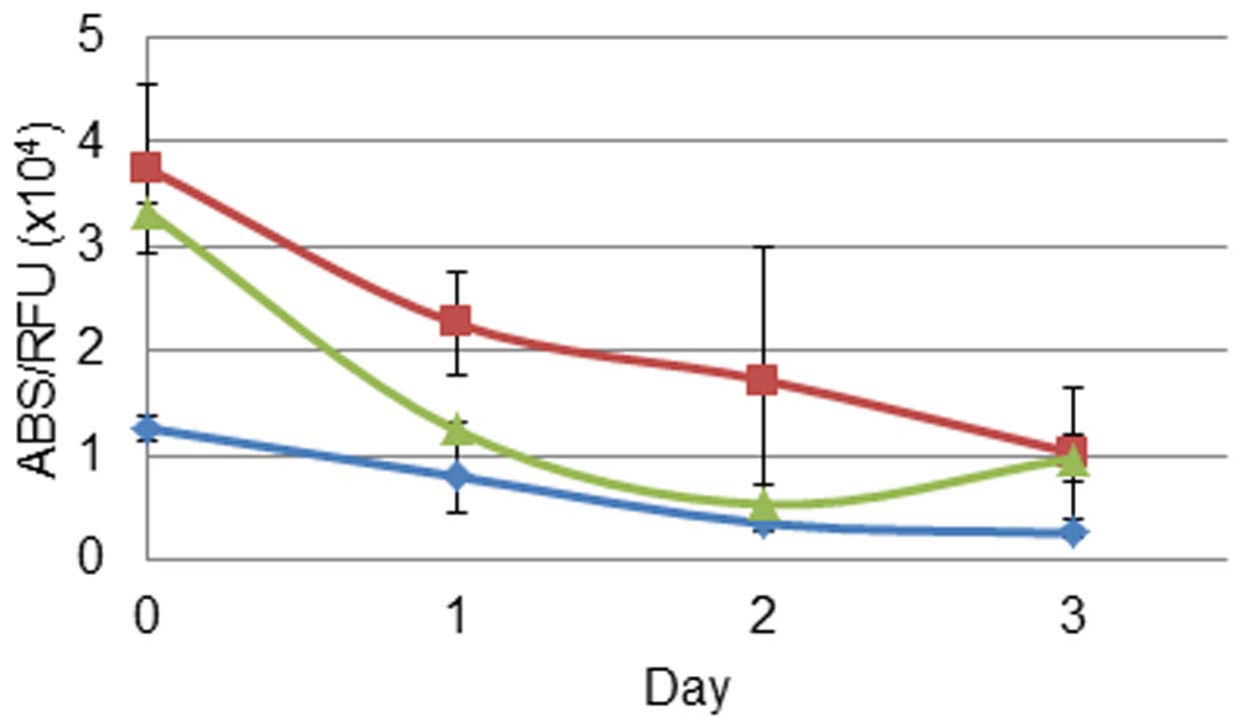
PNP-AV binding stability. Bound fusion protein over a 3-7 (♦), MDA-MB-231 (▴), and HAAE-1 (▪) cell lines. Data are presented as mean ± SE (*n = *3).

PNP-AV binding per cell was studied over 3 days for the three cell lines to determine the stability of binding. The binding assay data (absorbance at 450 nm) was divided by the Alamar Blue fluorescence data for cell viability (relative fluorescence at 530 nm excitation, 590 emission) (Fig. 4). Initially, the endothelial cells and MDA-MB-231 cancer cells had similar amounts of binding, while the amount for MCF-7 cells was less. For all three cell lines, the binding of the protein per cell declined over 3 days; however, binding was still measureable after 3 days.

**Figure 5:**
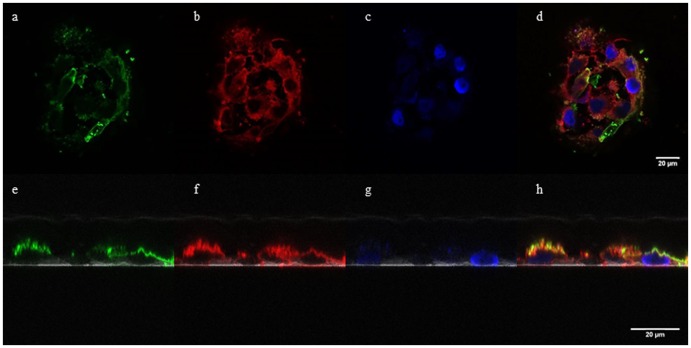
PNP-AV membrane binding visualization. Confocal microscopy of MCF-7 cells confirms the presence of externally bound biotinylated PNP-AV, with streptavidin-conjugated Alexa Fluor 488 in green (a, e), red CellMask stain of the plasma membrane (b, f), and blue Hoechst 33258 dye staining of nucleic acids (c, g). Images a-c show the color channels of an x-y confocal image with the composite shown in d. Images e-g show the color channels of an x-z cross-section of cells with the composite shown in h and the coverslip indicated by the white in each image.

To illustrate the binding of PNP-AV to the cell membrane, MCF-7 cells were fixed, incubated with biotinylated PNP-AV, and then incubated with streptavidin conjugated Alexa Fluor 488. Fig. 5 shows confocal microscopy images of the protein bound to the external surface of the cells with nuclei and plasma membrane counterstains. It is observed that the binding of PNP-AV (green) is found only on the plasma membrane (red) but does not completely cover the membrane.

### In vitro Cytotoxicity

**Figure 6:**
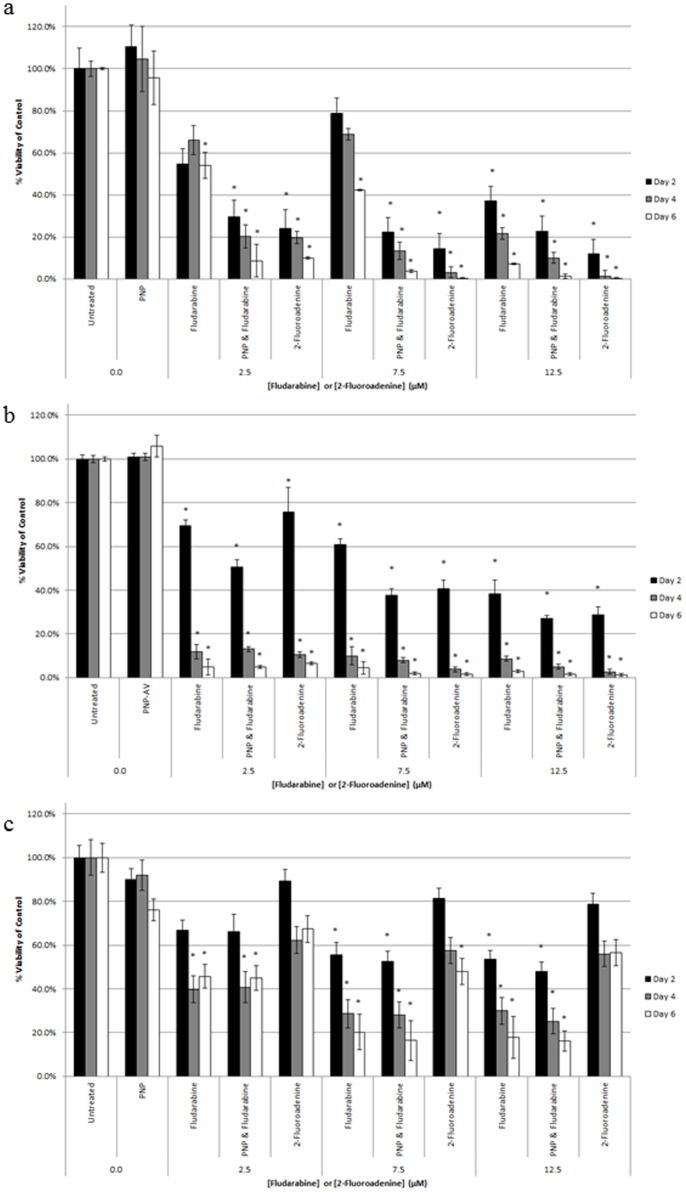
*In vitro* cytotoxicity of enzyme prodrug treatment. The effects of fludarabine, 2-fluoroadenine, and fludarabine converted to 2-fluoroadenine by PNP are shown for (a) non-confluent HAAE-1 cells, (b) MCF-7 cells, and (c) MDA-MB-231 cells. Groups that received PNP-AV were treated on days 0 and 3 of the study. Fludarabine and 2-fluoroadenine were administered daily. Viability was determined by the Alamar Blue assay on days 2, 4, and 6 (black, gray, and white bars, respectively), and each sample was represented as a percentage of untreated control on each day. Statistical analysis was performed with a one-way ANOVA test with data presented as mean ± SE (*n = *3). Statistical significance vs. untreated control on the same day is denoted by *(*p*<0.001).

The results of the cytotoxicity studies for MCF-7 and MDA-MB-231 breast cancer cells and non-confluent HAAE-1 endothelial cells are shown in Fig. 6. The cell viability data is shown as a percentage relative to the untreated control on days 2, 4, and 6. There was significant cell killing (*p*<0.001) by treatments for all three cell lines, with greater than 95% cytotoxicity after 6 days of treatment for the MCF-7 and non-confluent endothelial cells; more than 80% cytotoxicity was obtained for the MDA-MB-231 cells after 6 days. For the non-confluent endothelial cells, there was more cell killing for the treatment with PNP-AV/fludarabine and 2-fluoroadenine compared to treating with fludarabine only. For the two breast cancer cell lines, the cell killing by treatment with fludarabine only, PNP-AV/fludarabine, and 2-fluoroadenine were similar. Cytotoxicity was also performed using confluent endothelial HAAE-1 cells, which showed no significant effect on cell viability (*p*<0.001) for treatment with fludarabine only or PNP-AV/fludarabine (data not shown).

## Discussion

In this study we were able to produce a recombinant fusion protein consisting of *E. coli* purine nucleoside phosphorylase and human annexin V, while maintaining strong binding capability and good enzymatic activity. Recombinant PNP production in other studies has yielded enzyme activities between 27 and 180 U/mg [11]. The PNP-AV activity in the present study was determined to be 35 U/mg, which corresponds to an activity of 87.5 U/mg when accounting for only the PNP portion of the fusion and is consistent with the activity of recombinant PNP produced by other groups.

The *in vitro* binding studies presented in this paper confirm strong binding to the membranes of cancer cells and non-confluent vasculature endothelial cells. Endothelial seeding densities that lead to non-confluence result in the activation of the endothelial cells, including HAAE-1 cells, which models the tumor vasculature [31]. Earlier studies in our laboratory have shown that annexin V in a fusion protein binds to non-confluent endothelial cells but does not bind to these cells when they are confluent [21], which means that non-confluent endothelial cells mimic the PS exposure of the tumor vasculature; this is consistent with the finding by others that annexin V binds to phosphatidylserine expressed on endothelial cells of tumor vasculature, but not normal endothelial cells [19], [20]. The lack of binding of the PNP-AV fusion protein to confluent endothelial cells was further confirmed in this study when we found that a *K_d_* value for specific binding could not be obtained, thus indicating that confluent endothelial cells mimic the normal vasculature.

The PNP-AV binding was found to be significantly stronger than AV and AV fusions with cytosine deaminase and L-methioninase [21], [22], possibly because PNP-AV is a hexamer with six annexin V’s present on each protein; cytosine deaminase and L-methioninase are a dimer and a tetramer, respectively. Modeling of the structure of the fusion protein, shown in Fig. 2, suggests that the annexin V components of the fusion are accessible and available for binding. Confocal microscopy confirms that the fusion protein is bound to the cell membrane but does not continuously cover the membrane (Fig. 5). This type of discontinuous exposure of PS has also been observed by others for MDA-MB-235 breast cancer cells [32].

This study indicates a significant cytotoxic effect of this enzyme prodrug system on breast cancer cell lines MCF-7 and MDA-MB-231 and endothelial cells representative of the tumor vasculature, non-confluent HAAE-1. A 6-day incubation period is clearly relevant for the enzyme prodrug treatment for all three cell lines tested, since the cell viability declined after each 2-day interval in every case. The enzyme prodrug treatment was particularly effective for the MCF-7 cancer cells and the non-confluent endothelial cells, where greater than 95% cytotoxicity was obtained after 6 days of treatment. The lack of significant effect on confluent endothelial cells is a further indication that no binding of the fusion protein had occurred and that confluent endothelial cells are representative of the normal vasculature; this is consistent with our previous finding that the cytosine deaminase-annexin V fusion protein did not bind to confluent endothelial cells [21].

The *in vitro* cytotoxic effect of fludarabine alone on breast cancer cells confirms work in another study that found significant killing between 2.5 and 10 µM fludarabine, particularly with the MCF-7 cell line [3]. This could potentially result from active transport across the cell membrane and an increased capacity to retain nucleoside analogue triphosphates in tumor cells (see Fig. 1) [33]. Despite the promising results of fludarabine alone, a phase II clinical trial suggests that this strategy is not sufficient for clinically advanced breast cancers [34]. Myelosuppression was the major dose-limiting factor that held fludarabine below effective levels. The enzyme prodrug approach is a potential improvement over fludarabine treatment, as the conversion of fludarabine to 2-fluoroadenine increases the compound’s toxicity, affects dividing and non-dividing cells, and allows for a bystander killing effect due to free diffusion across membranes [10]. Thus, intensification of the cytotoxic effect only at the site of the tumor would allow for a reduced systemic dose of fludarabine while enhancing treatment efficacy.

Following administration of the PNP-AV fusion protein in a clinical treatment, an immune response may occur because of *E. coli* PNP being present. To avoid this problem, a human PNP could be used that has been mutated with two amino acid substitutions so that it can utilize fludarabine as a substrate [35]. Another approach would be to conjugate the *E. coli* PNP with polyethylene glycol (PEG) to reduce or eliminate an immune response; PNP from *E. coli* has been successfully PEGylated to substantially reduce its immunogenicity while maintaining enzyme activity [36]. Human AV is not expected to induce an immunogenic response.

In conclusion, we were able to produce a recombinant fusion protein of PNP-AV that binds and kills breast cancer cells and endothelial cells that mimic the PS exposure of tumor vasculature when used in an enzyme prodrug therapy with fludarabine. The results suggest that this treatment could be useful as a targeted therapy for breast cancer.
